# Patients with multiple sclerosis: a burden and cost of illness study

**DOI:** 10.1007/s00415-022-11169-w

**Published:** 2022-05-23

**Authors:** Mario Alberto Battaglia, Daiana Bezzini, Isabella Cecchini, Cinzia Cordioli, Francesca Fiorentino, Tommaso Manacorda, Mihaela Nica, Michela Ponzio, Daniela Ritrovato, Chiara Vassallo, Francesco Patti

**Affiliations:** 1 Italian Multiple Sclerosis Foundation (AISM), Genoa, Italy; 2 The University of Siena, Siena, Italy; 3 IQVIA Solutions Italy S.r.l., Milan, Italy; 4 ASST Spedali Civili di Brescia, Multiple Sclerosis Center, Brescia, Italy; 5 The London School of Hygiene and Tropical Medicine, London, UK; 6 Novartis Farma S.p.A., Origgio, Italy; 7 Multiple Sclerosis Centre Sicilia Region, University Hospital Catania, Catania, Italy

**Keywords:** Multiple sclerosis, Burden of illness, Cost of illness, Healthcare costs, Payer, Italy

## Abstract

**Background:**

Multiple sclerosis (MS) is a chronic neuroinflammatory and neurodegenerative disease negatively impacting patients’ physical, psychological and social well-being with a significant economic burden.

**Objectives:**

The study estimates MS burden and cost of illness in Italy from a societal perspective in 2019.

**Methods:**

Information on the impact of the disease on daily activities, symptoms, employment, resource utilization and the role of caregivers was collected through questionnaires completed by 944 patients and caregivers. Results were stratified according to both disease severity and payer. Mean costs and overall costs were extrapolated from the sample to the Italian MS population considering published distribution of severity.

**Results:**

The study showed a great impact of the disease on daily and work activities increasing with the disability. The overwhelming burden of fatigue emerged. Mean annual costs were estimated at €39,307/patient (€29,676, €43,464 and €53,454 in mild, moderate and severe cases, respectively). Direct healthcare costs were the major component (€21,069), followed by indirect costs (€15,004). The overall cost of the disease in Italy was €4.8 billion. The National Healthcare System (NHS) sustained most of the costs (80%), most notably direct healthcare costs, while patients paid almost all non-healthcare expenses.

**Conclusions:**

This study confirmed that MS carries a substantial burden to patients and society, highlighting the need for awareness of this disease.

**Supplementary Information:**

The online version contains supplementary material available at 10.1007/s00415-022-11169-w.

## Introduction

Multiple sclerosis (MS) is a chronic and neurodegenerative disease, in which the immune system’s abnormal response damages the central nervous system [1].

Recent estimates hypothesize 2.8 million MS cases worldwide [2], while in Italy, epidemiology evaluations report over 122,000 patients in 2019 with an annual incidence of 3400 new cases [3].

MS development can usually be classified in four phenotypes: radiologically isolated syndrome (RIS)/clinically isolated syndrome (CIS), initial courses; relapsing remitting (RRMS), the most common type (85% of diagnoses); secondary progressive (SPMS), an evolution of RRMS affecting 65% of RRMS patients; primary progressive (PPMS), a rare and purely progressive type [1].

New classifications [1] also classify the disease between relapsing (RRMS and SPMS with relapses) and progressive disease (PPMS and SPMS without relapses).

MS is often disabling, leading to a wide range of symptoms, such as loss of vision, ataxia, tremor, bowel incontinence and/or urinary incontinence, generalized pain, fatigue, memory and learning problems, depression and anxiety [4]. Initial symptoms often appear between 20 and 40 years of age [3]. Due to the wide range of manifestations, their debilitating nature and their onset during patients’ most active and productive years, MS has an enormous impact on patients’ physical, psychological, social and economic well-being. Indeed, while MS causes a progressive reduction in patients’ physical and cognitive functions until patients need continuous assistance, the effect on their lifespan is limited [5]. This causes a high economic burden of MS on society, especially in Italy, where the role of unpaid caregivers remains central in disease management [6]. For these reasons, the estimation of the MS socio-economic burden has been of particular importance in the last 20 years in Italy [6–10].

The objective of this study is to contribute to and update the existing literature estimating the MS burden on patients’ life and on society, according to different disease severities. This is the first analysis specifically detailing who the relevant payers are according to different cost categories.

## Materials and methods

### Study design

The study was designed using a bottom-up approach, collecting information from a sample of 873 patients with MS and 71 caregivers across Italy, identified by the Italian Multiple Sclerosis  Foundation (Associazione Italiana Sclerosi Multipla, AISM).

AISM contacted its members to participate in the study through the completion of a Computer Assisted Web Interviewing (CAWI) questionnaire. The questionnaire was designed for this specific purpose after a literature review of other European cost of illness studies in the therapeutic area and 20 qualitative interviews with patients. The questionnaire was then validated by two Italian clinical experts, AISM and two patients. In particular, the clinical experts and the association validated the relevant information to assess MS burden and the presence of all relevant elements to estimate its costs in the Italian context. Afterwards two pilot interviews with patients were organized before the official beginning of the questionnaire to assess the questionnaire’s clarity.

Interviews were conducted from May 2021 to July 2021. Two versions of the questionnaire were developed, one for patients and one for caregivers. A unique link to the questionnaire was provided to each participant. By accessing the link, the patients or caregivers were first asked to express their informed consent regarding their anonymous participation in the study.

The analysis was in reference to the year 2019, so to avoid possible biases in results due to the effect of the CoViD-19 pandemic and resulting lockdowns in 2020 and 2021, patients and caregivers were hence asked to refer specifically to the year 2019 when reporting MS resources consumption and expenditures.

In this analysis, patients were stratified in three groups according to their Expanded Disability Status Scale (EDSS) score [11], being mild MS (EDSS ≤ 3.5), moderate MS (EDSS: 4–6.5) and severe MS (EDSS > 6.5).

All results are presented per average patient with MS, weighted considering the distribution of severity reported in the literature [8] (41% patients with an EDSS score ≤ 3.5, 44% patients with an EDSS score between 4 and 6.5 and 15% patients with an EDSS score > 6.5). Moreover, results are shown by disability group (mild, moderate and severe) and by payer (i.e., who ultimately is responsible for the expenditure among the National Healthcare System, NHS [Sistema Sanitario Nazionale, SSN], patients themselves and their families [out of pocket expenditure] or third-party [insurances, non-profit organizations]).

Costs were extrapolated from the sample to the Italian MS population based on the most recent prevalence estimate [3].

### Patient characteristics and burden of illness

We collected patients’ socio-demographic and clinical information. Socio-demographic data included age, gender, residence, level of education, employment and income information, presence of one or more caregivers. Clinical data included EDSS score, multiple sclerosis type, age at diagnosis and at appearance of first symptoms.

To investigate the burden of MS on quality of life, patients were asked to self-assess their disability using descriptions based on the EDSS, major symptoms and MS impact on work and day-to-day life.

### Cost of illness

The cost of MS in Italy was calculated for the year 2019 (expressed in 2019 Euro), considering a societal perspective, thus including direct healthcare costs, direct non-healthcare costs and indirect costs in terms of productivity and income loss and lost leisure time [12].

Direct costs included:

- healthcare costs: hospital admissions, rehabilitation at home, day hospital and outpatient visits at MS centers, additional outpatient medical visits (GPs, specialists, psychologists, osteopaths, acupuncturists, masseurs, other physical therapists), tests and diagnostic procedures, pharmacological treatments and external technical aids/orthoses (see Supplementary Material);
- non-healthcare costs: transport, paid assistance (nurses, caretakers, domestic help), house and car modifications due to MS (see Supplementary Material).

Direct unit costs for each identified resource were obtained or estimated based on national tariffs [13], regional [14–18] or hospital [19–23] sources and published literature [24] and, when relevant, were inflated to 2019 prices [25]; out of pocket and third-party expenditures were mostly reported directly by the patients or their caregivers.

Drug costs were calculated at market ex-factory prices net of lawful discounts and were based on official posology from AIFA Summary of Product Characteristics [26, 27]. When the brand name was not reported, the median price of the alternatives was considered. Disease-modifying therapies (DMTs) costs were calculated yearly, while symptomatic were assumed to be taken only for one cycle. Details on additional direct unit costs used in the study are reported in Supplementary Material.

Indirect costs included both patients and caregivers’ productivity losses due to MS, based on the Human Capital Approach [28], as well as caregivers’ leisure time lost while taking care of patients.

Details are reported in Table 1.

**Table 1:** Indirect costs included in the analysis

Patient/caregiver category	Cost category	Description
Employed patient	Presenteeism	Productivity loss caused by MS impact on time spent at work in terms of quality and quantity of work done: valued multiplying gross salary and patients’ declared lack of productivity due to MS^a^ expressed as percentage
	Absenteeism	Productivity loss due to hours of leave: valued multiplying number of hours lost due to MS (exams, visits, etc.) and the gross salary per hour, net of presenteeism effect
	Salary decrease	Productivity loss caused by a decrease in salary due to MS: valued as difference between previous and current gross annual salary, when reduction was reported as associated to MS
Inactive patients	Lower participation to the job market	Productivity loss due to due to patients’ inactivity: valued as gross yearly salary before leaving job due to MS
Studying patients	Delayed entrance in the job market	Productivity loss caused by studying patients’ lower employment due to MS: valued considering the annual gross salary^b^ multiplied by the employment rate^c^ of an average person with the same socio-demographic characteristics (in terms of sex and age group)
Retired patients	Early retirement	Productivity loss caused by early retirement: valued considering the patients’ gross yearly salary before retirement for retired patients 65 years old or younger
Employed caregivers	Absenteeism	Productivity loss due to hours of work leave (Legge 104/92)^d^ to support MS patients: valued multiplying the gross salary per hour of an average person with the time lost for accompanying the patients to exams, visits, etc
	Leisure time dedicated to patients	Caregivers’ leisure time lost taking care of patients with MS: valued from net salary per hour multiplied by the number of hours dedicated to patients, with a cap of 8 h per day maximum^e^
Inactive caregivers	Lower participation to the job market	Productivity loss caused by caregivers’ early exit from the job market because of patients’ MS requiring caregivers’ support: valued as caregivers’ gross yearly salary before leaving job
	Leisure time dedicated to patients	Caregivers’ leisure time lost taking care of patients with MS: valued from average net disposable income in Italy^b^ per hour multiplied by the number of hours dedicated to patients, with a cap of 8 h per day^7^

Methodological note: when present, the salary self-reported by the patients or by the caregiver was used; when not reported, if feasible, the reported average salary of patients or caregivers with the same socio-demographic characteristics (in terms of gender and age cluster) was used; finally, if salary was not reported neither for the patient (caregiver) nor for the patients (caregivers) with the same socio-demographic characteristics, ISTAT data were used^b^

*MS* multiple sclerosis

^a^Work Productivity and Activity Impairment Questionnaire: General Health (WPAI:GH); Italian version

^b^JP Salary Outlook 2020. L’analisi del mercato retributivo italiano

^c^Tasso di occupazione—dati trimestrali destagionalizzati. I.stat, http://dati.istat.it/Index.aspx?QueryId=23244# (Accessed 5 March 2021)

^d^INPS, https://www.inps.it/prestazioni-servizi/indennita-per-permessi-fruiti-dai-lavoratori-per-assistere-familiari-disabili-in-situazione-di-gravita-o-fruiti-dai-lavoratori-disabili-medesimi (Accessed 2 December 2021)

^e^Battaglia et al. [7]

Disability pensions and accompanying allowances were not included in the cost estimate, since they are financial transfers from national pension institutions (Italian INPS and INAIL) and do not involve a use of resources [6]. However, since this information might be of interest to policy makers, transfers’ values were reported.

### Statistical analysis

The analysis was carried out with Stata version 11 software.

Descriptive statistics were performed to investigate patients’ socio-demographic and clinical characteristics, resource utilization and costs.

In the cost of illness analysis, a non-parametric Kruskal Wallis statistical test was applied to compare the group means and to determine whether differences were statistically significant (*p* value < 0.05 for significance). In addition, differences in costs (total, direct and indirect) among educational levels (primary, diploma and graduated or higher) and income groups were tested.

Also, four scenario analyses were conducted to test the robustness of base case results and to evaluate the more uncertain parameters of this analysis, particularly, self-reported severity, EDSS relative weight for results extrapolation, relevance of non-disease modifying drugs for MS and, lastly, adherence to DMTs.

The first scenario assessed the impact on the results considering the most severe EDSS score between the self-assessed disability, using descriptions based on the EDSS, and that stated in their medical records (which was considered in the baseline analysis).

In the second scenario, results from the sample to the general MS population were extrapolated according to EDSS score distribution from a different source (the Global Burden of Disease, 2016 [29]). In the third scenario, all the non-DTMs were excluded, making the extreme assumption that all of them were for comorbidities only and not for MS symptoms. Finally, in the fourth scenario, it was assumed that patients were 80% adherent to the treatment with DMTs, while in the baseline, we considered patients adherent for the entire year.

## Results

Nine hundred and forty-four evaluable responses were received; in the cost of illness, we included only information reported by or relating to patients diagnosed before 2020, therefore, 9 observations where diagnosis of MS was reported in 2020 were dropped, leaving a sample of 935 responses.

In the studied sample, 64% of patients were women and 36% men, with a mean of 49 years of age (Standard Deviation, SD: 11 years). The majority of patients were from the North–West of Italy (34%), followed by the North–East (23%), Central Italy (26%) and Southern Italy and the Islands (17%). With regards to the educational and employment level, more than half of the patients had a high school diploma and 36% held an undergraduate or higher university degree; almost 60% patients were employed, 16% were unemployed, 23% were retired and 2% were students.

About 40% of patients were supported by at least one caregiver.

### Patient characteristics and burden of illness

Data on patient characteristics and their burden according to EDSS status are summarized in Table 2.

**Table 2:** Patients’ characteristics and burden of illness

	Mild MS EDSS ≤ 3.5	Moderate MS EDSS = 4–6.5	Severe MS EDSS > 6.5	Total patients (extrapolated data)
No. of observations	540	265	130	122,100
Men (% of patients)	27%	41%	48%	36%
Mean age of responders (years)	42	53	56	49
Mean age at diagnosis (years)	32	37	36	35
Mean age at first symptoms (years)	29	33	31	31
Level of education (% of patients)				
Primary	8%	12%	21%	12%
Diploma	50%	56%	49%	52%
Graduated or higher degree	42%	32%	31%	36%
Type of MS (% of patients)				
Primary progressive (MS-PP)	6%	21%	25%	15%
Secondary progressive (MS-SP)	2%	30%	61%	23%
Relapsing–remitting (MS-RR)	89%	48%	14%	60%
Clinical isolated syndrome (CIS) or radiologically isolated syndrome (RIS)	4%	1%	0%	2%
MS impact on every-day life (% of patients)				
Highly	7%	36%	78%	29%
Quite highly	23%	46%	17%	33%
Moderately	31%	14%	4%	20%
A little	30%	4%	1%	15%
Not at all	9%	0%	0%	4%
Symptoms impacting patients’ every-day life the most (% of patients)				
Fatigue	70%	75%	57%	70%
Equilibrium	16%	50%	34%	34%
Urinary problems	19%	37%	47%	31%
Impaired sensitivity	15%	12%	10%	13%
Memory and concentration problems	26%	16%	9%	19%
Spasticity	4%	21%	45%	17%
Pain	8%	18%	19%	14%
Intestinal problems	6%	9%	16%	9%
Visual problems	10%	7%	9%	9%
Main aspects impacting quality of life (% of patients)				
Physical fatigue	60%	84%	85%	74%
Impact on life plans	52%	74%	84%	65%
Physical difficulties	26%	84%	97%	61%
Difficulties in moving and traveling	19%	69%	86%	50%
Social and relational life	28%	60%	77%	49%

*MS* multiple sclerosis, *EDSS* expanded disability status scale

In our sample, more than half of patients (58%) were affected by a mild disease, 28% by a moderate disease, while the group of severe patients was the smallest one (14%). The majority of patients (60%) had RRMS, but as the MS disability worsens the proportions shift towards SPMS and PPMS.

Fatigue was both the most common symptom, present in 70% of patients, and the one with the greatest impact on their lives, regardless of the disease disability level, followed by balance issues (34% of patients) and urinary problems (31%).

More than 60% of patients asserted that MS impacts quite highly or highly on their life; in particular, main complaints were physical fatigue, impact on life-plans, difficulties in moving and traveling and the negative effect on one’s social life.

Among the 58% of employed patients, 71% asserted that MS had a negative impact on their work productivity, 65% lost on average 4.8 working hours per week and 15% suffered a salary decrease. Some patients had to prematurely leave the job market or delay their entrance: more than half of the 16% of unemployed patients left their jobs due to the disease and 13% of students (2% of patients) were finishing their studies late.

Moreover, among the 59% of employed caregivers, 29% reported asking for hours of leave to assist patients during work hours, and among the 7% of unemployed caregivers, 20% quit the job due to the patient’s disease.

### Cost of illness

**Fig. 1:**
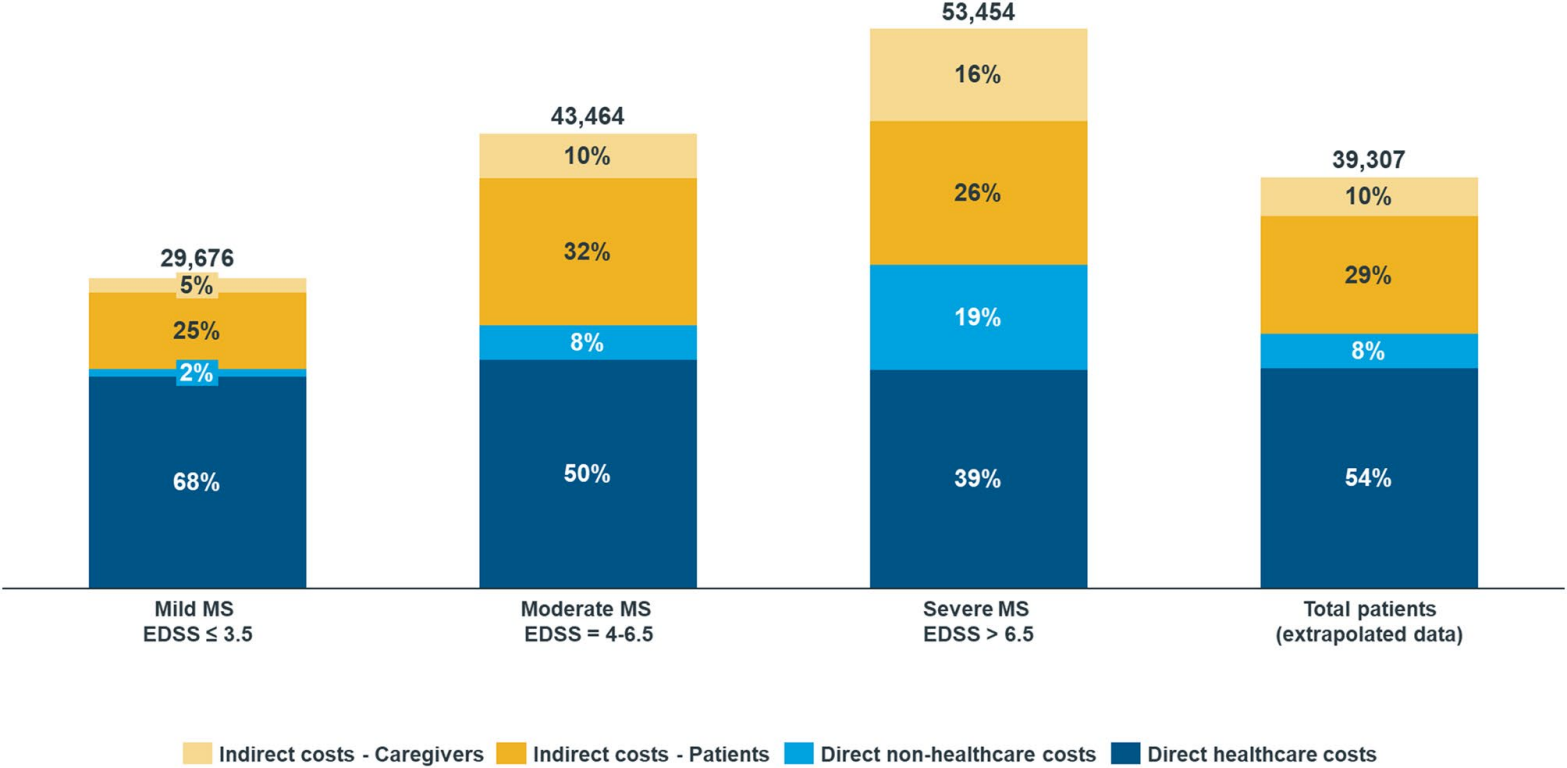
Mean annual cost per patient by disability level in 2019

Total costs associated with MS are presented in Table 3 and in Fig. 1.

**Table 3:** Mean annual cost per patient by disease severity in 2019

Type of cost	Mild MS EDSS ≤ 3.5 Mean (SD)	Moderate MS EDSS = 4–6.5 Mean (SD)	Severe MS EDSS > 6.5 Mean (SD)	Total patients (extrapolated data) Mean (SD)	*P* value^a^
Total costs	29,676€ (15,050€)	43,464€ (24,325€)	53,454€ (29,397€)	39,307€ (23,583€)	< 0.01
Direct healthcare costs	20,253€ (9,673€)	21,869€ (12,420€)	20,915€ (14,377€)	21,069€ (11,719€)	< 0.05
Hospitalizations	199€ (1,494€)	441€ (1,555€)	741€ (2,092€)	387€ (1,630€)	0.107
Rehabilitation at home	26€ (193€)	199€ (622€)	647€ (993€)	194€ (610€)	< 0.01
Day hospital/outpatients	1,975€ (3,964€)	2,963€ (5,078€)	2,478€ (5,662€)	2,489€ (4,768€)	< 0.05
Extra visits	63€ (188€)	115€ (286€)	137€ (298€)	97€ (254€)	< 0.05
Exams	403€ (273€)	435€ (339€)	296€ (328€)	402€ (315€)	< 0.01
Therapies	17,549€ (8,304€)	16,570€ (10,082€)	12,826€ (10,894€)	16,419€ (9,643€)	< 0.01
External aids/orthoses	39€ (204€)	1,146€ (2,102€)	3,790€ (3,839€)	1,083€ (2,378€)	< 0.01
Direct non-healthcare costs	731€ (1,599€)	3,301€ (9,878€)	9,979€ (17,061€)	3,234€ (9,803€)	< 0.01
Transport	253€ (595€)	327€ (620€)	379€ (925€)	304€ (665€)	< 0.01
Paid assistance	371€ (1,187€)	1,341€ (2,682€)	6,110€ (9,002€)	1,646€ (4,386€)	< 0.01
Car/house modification	107€ (813€)	1,633€ (9,394€)	3,489€ (15,288€)	1,283€ (8,654€)	< 0.01
Indirect costs	8,692€ (11,186€)	18,294€ (16,842€)	22,561€ (15,093€)	15,004€ (15,481€)	< 0.01
Patients’ indirect costs	7,272€ (10,064€)	14,042€ (15,227€)	13,791€ (12,363€)	11,243€ (13,320€)	< 0.01
Caregivers’ indirect costs	1,420€ (3,792€)	4,252€ (6,693€)	8,770€ (8,443€)	3,760€ (6,495€)	< 0.01

*MS* multiple sclerosis, *EDSS* Expanded Disability Status Scale, *SD* standard deviation

^a^Significancy of difference between disability levels was tested through non-parametric Kruskal Wallis statistical test; *p* value < 0.05 was used for significance

Average cost per MS patient per year from a societal perspective was estimated at €39,307 (SD: €23,583), from €29,676 (SD: €15,050) in patients with mild disease to €53,454 (SD: €29,397) in patients with severe disease. Costs differences among disability levels (mild, moderate and severe) were statistically significant.

Overall costs were significantly different among different income groups, while indirect costs were found significantly different among both educational and income groups.

Contribution of direct healthcare costs, direct non-healthcare costs and indirect costs to overall costs was estimated at 54%, 8% and 38%, respectively.

Direct healthcare costs were estimated at €21,069 (SD: €11,719), with pharmacological treatment accounting alone for 78% of them.

Pharmacological treatment costs had a relevant weight on overall costs (42%). Their relative contribution decreased with increasing disease severity (81% and 61% of direct healthcare costs in mild and severe patients respectively) and they were almost completely associated to DMTs (96%).

Annual direct non-healthcare costs were estimated at €3,234/patient (SD: €9,803) with a high variation among severity groups; the most impactful direct non-healthcare cost was represented by paid assistance, especially in patients with an EDSS higher than 6.5.

Average patients’ indirect costs per year were estimated at €15,004 (SD: €15,481). Overall productivity loss per employed patient was evaluated at over €5,000, of which 70% driven by presenteeism. Early exit from and delayed entrance into the job market accounted overall for an average cost of about €2,400. Early retirement costs were valued equal to €3,600. Considering caregivers’ indirect costs, leisure time lost had the heaviest impact, €3,330 per patient on average.

We estimated disability pension values: on average MS patients received almost €1,800 per year as allowances, varying from €450 (SD: €1,937) in mild patients to €3,872 (SD: €2,858) in severe patients.

**Fig. 2:**
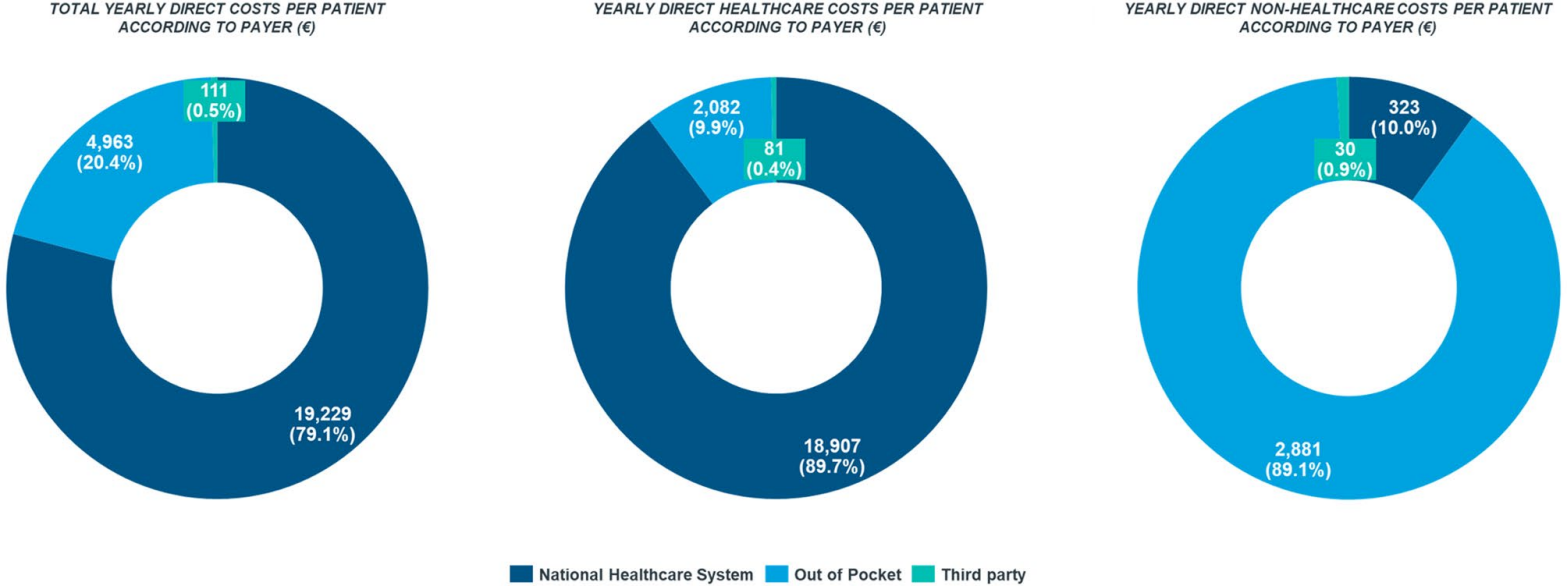
Direct multiple sclerosis costs according to main payer

Details on main payers in MS are reported in Fig. 2.

In terms of cost distribution by payer (NHS, patients and their family and third-party), almost 80% of MS costs were financed by the NHS. Among direct healthcare costs, the NHS sustained the great majority of costs (90%, €18,907 [SD: €10,838]); while almost all direct non-healthcare expenses were paid directly by patients or their families, corresponding to almost €5,000/patient annually.

Costs impacting the majority of patients and their families’ finances were: paid assistance, house renovation and car modification, non-DMTs and external aids, with a relative weight of 27%, 25%, 15% and 13%, respectively. Overall, third-party payers contributed to less than 1% of overall costs.

### Scenario analyses

In the scenario analyses performed, the overall annual burden of MS varied between 36,159€ (− 8%) and 38,809€ (− 1%) showing a robustness of the results.

In particular, the first scenario, where the most severe EDSS score between the one was asked to be retrieved from the patient’s medical records and the self-assessed disability descriptions, was the least impacting scenario with a decrease of overall burden of approximately 1% (− 498€).

The second least impacting scenario is the third one where costs related to the drugs different from DMTs were excluded. Here, overall burden decreases by 2% (− 680€).

In the second scenario, where results to the general MS population were extrapolated based on a different source [29], and the fourth scenario, where 80% adherence to the treatment with DMTs was considered, results varied by 7% (− 2,867€) and 8% (− 3,148€), respectively.

## Discussion

Multiple sclerosis is a neurological disease that carries a substantial physical, personal, social and economic burden, which increases with disease severity.

Our results highlighted that patients with MS are affected by several burdensome and disabling symptoms, above all the overwhelming presence of fatigue. We also underlined that multiple sclerosis has a negative impact on patients’ daily life, in terms of life-plans, difficulties in traveling, on their social life, and on their work, in terms of attention and presence at work, salary and early exit from the job market or retirement.

The cost of illness analysis showed that MS costs in 2019 accounted for €39,307/patient and for €4.8 billion for the entire Italian society, corresponding to 0.3% of the national GDP [30]. Overall annual costs per patient increased with disability, varying from €29,676 for mild disease to €53,454 in severe cases. The main cost component was direct healthcare costs, followed by indirect costs.

To the best of our knowledge, the present cost of illness study included two original findings. The first one regarded main payers of MS: more than three quarters of costs were sustained by the NHS, especially direct healthcare costs, while almost all non-healthcare expenses were paid directly by patients; the second one regarded the investigation of presenteeism in MS, evaluated for the first time.

The bottom-up approach allowed us to include all relevant costs both within and outside the health care system and to assess the correlation with disease severity. In addition, the present study was based on a questionnaire designed specifically and validated by AISM and clinicians to estimate the burden and cost of MS specifically in the Italian context.

Moreover, comparing this analysis with the available literature, the methodology and results were in line with other published studies on MS in the Italian setting, in terms of physical, psychological and economic burden.

In fact, also Battaglia et al. [7] highlighted that MS affected productivity at work, that almost all the patients experienced fatigue and cognitive difficulties and that multiple sclerosis has a negative impact on patients’ life, especially in the most severely affected patients.

Also, results of Italian cost of illness studying the economic burden of MS from a social perspective [7–9], inflated at 2019 [25] and extrapolated with the same weightings used in the present analysis [8], ranged from €36,400/patient per year in Battaglia et al. [7] to €37,700 in Karampampa et al. [9] and to €40,600 in Ponzio et al. [8] and in all cited studies costs increased with increasing disability.

Cost category splits could be compared with earlier studies as well, both in percentage and absolute values [7–10], with a notable similarity to Battaglia et al. [7]: in the present study, the largest cost component was direct healthcare cost, accounting for 54% (60% in Battaglia et al. [7]), followed by indirect costs, accounting for 38% (34% in the cited study).

Interestingly, if on the one hand, macro-cost components relative weights were similar to previous studies, on the other hand, the single cost items were subject to variation. Particularly, in the present study, DMTs costs were higher than the most recent estimates [7] and this could be explained by two factors: the recent introduction of high-cost drugs [26] and the assumption made in our analysis that all patients were completely adherent to DMTs, dictated by the structure of the questionnaire (to address this possible concern we performed a scenario analysis assuming a lower treatment adherence).

In addition to assumptions related to DMTs adherence, our study presents some other limitations.

First, the study sample represented 1% of total MS population with an under-representation in Southern Italy; in any case, the population size was in line with or higher than existing literature samples [6, 7, 9, 10] and we believe that it was adequate to provide informative results.

Second, the patients and caregivers who participated in the study had to be enrolled in the patient association and participated voluntarily. This recruitment process may have led to some selection bias, nevertheless, it is a common methodology in published cost of illness studies in Italy [7, 8].

Third, our study was based on patients’ self-reported responses to a questionnaire: answers could thus be affected by a certain degree of subjectivity and some resource consumption could be at least partly attributable to other diseases [10]. To limit this risk, we validated our questionnaire with an advisory board composed by two Italian clinicians and AISM and we specified in each question its pertinence to MS. Also, we tested one uncertain parameter that could greatly impact results, that being, patients’ declared EDSS score, with an impact on results of only 1%.

Fourth, our analysis possibly led to a recall bias [31]: the analysis was set for 2019 to prevent the health emergency due to CoViD-19 pandemic from impacting the results and not accurately representing the real context of MS.

Moreover, to extrapolate results from the general population, we referred to the source, that in our belief, best represents the Italian context [8], however, we are aware that different possible weights to base the extrapolation on do exist: therefore, we performed a scenario analysis, and thus showing a similar magnitude in results.

Finally, the collection and estimation of unit costs relied also on official national and regional tariffs, that might not reflect real costs. However, this usually represents the best practice in cost of illness studies [12].

To conclude, we believe that our analysis provides a valuable contribution in updating existing literature on the burden and cost of illness of multiple sclerosis in Italy. In addition, our study is the first to evaluate MS expenditure according to payers and presenteeism relating to work among MS patients’ indirect costs.

## Supplementary Information

Below is the link to the electronic supplementary material.

Supplementary file1 (DOCX 47 KB)
