# Supplementary material for: Patients with multiple sclerosis: a burden and cost of illness study
Source: J Neurol. 2022 May 23;269(9):5127–35. doi: 10.1007/s00415-022-11169-w (PMC9124746; doi:10.1007/s00415-022-11169-w)
Supplement: Supplementary file 1 — Supplementary file1 (DOCX 47 KB) [file 415_2022_11169_MOESM1_ESM.docx]

**Patients with multiple sclerosis: a burden and cost of illness study**

Journal of Neurology

Mario Alberto Battaglia, Daiana Bezzini, Isabella Cecchini, Cinzia Cordioli, Francesca Fiorentino, Tommaso Manacorda, Mihaela Nica, Michela Ponzio, Daniela Ritrovato, Chiara Vassallo, Francesco Patti

**Corresponding author**

Chiara Vassallo

E: chiara.vassallo@iqvia.com

M: +39 333 932 6974

IQVIA Solutions Italy S.r.l.
Via Fabio Filzi 29
20124 Milano
Italia

# **Supplementary material**

SUPPLEMENTARY MATERIAL 1: DETAILS ON ADDITIONAL DIRECT HEALTHCARE COSTS

| **External aids and orthosis** | | |
| --- | --- | --- |
| Item | Cost (€) | Description |
| Adult diapers/incontinence pads | 0.52 € | Lowest single diaper commercial price^1^ |
| Arm braces | 200.00 € | Contribution for braces to treat bone fractures or ligament lesions^2^ |
| Communication aids; kitchen aids; eating aids; writing aids; personal hygiene aids | 1,924.93 € | “Special utensils/devices”, mean annual cost per user inflated to 2019^3,4^ |
| Crutches/crane | 150.87 € | “Walking aids”, mean annual cost per user inflated to 2019^3,4^ |
| Electric wheelchair/scooter | 2,167.20 € | “Electric wheelchair, scooter”, mean annual cost per user inflated to 2019^4,5^ |
| External catheter | 25.88 € | Average of urethral catheter tariffs found in Regional Tariffs^6–8^ |
| Glasses | 246.82 € | Mean annual cost per user inflated to 2019^4,5^ |
| Leg orthosis/braces | 200.00 € | Contribution for braces to treat bone fractures or ligament lesions^2^ |
| Manual wheelchair | 1,819.00 € | Mean annual cost per user inflated to 2019^3,4^ |
| Mechanical bed (bed lift) | 7,423.86 € | Mean annual cost per user inflated to 2019^4,5^ |
| Orthopedic shoes | 100.00 € | Insurance contribution for orthopedic adult shoes^2^ |
| Tilting wheelchair | 1,819.00 € | “Wheelchair”, mean annual cost per user inflated to 2019^3,4^ |
| Walking frame | 150.87 € | Mean annual cost per user inflated to 2019^3,4^ |

1. Trovaprezzi.it, https://www.trovaprezzi.it/prezzi_ausili-disabili.aspx (accessed 24 November 2021).

2. FASDAC (Fondo Assistenza Sanitaria Dirigenti Aziende Commerciali). Nomenclatore Tariffario 2019.

3. Ponzio M, Gerzeli S, Brichetto G, et al. Economic impact of multiple sclerosis in Italy: focus on rehabilitation costs. *Neurol Sci* 2015; 36: 227–234.

4. Indice dei prezzi al consumo per famiglie operai e impiegati Gennaio 1947 - Ottobre 2021. *Istat Rivaluta*, http://rivaluta.istat.it:8080/Rivaluta/ (accessed 24 November 2021).

5. Kobelt G, Berg J, Lindgren P, et al. Costs and quality of life of multiple sclerosis in Italy. *Eur J Health Econ* 2006; 7: 45–54.

6. Regione Lombardia. Nomenclatore Tariffario Regionale per le prestazioni ambulatoriali.

7. Regione Marche. Nomenclatore Tariffario Regionale delle prestazioni specialistiche ambulatoriali.

8. Regione Lazio. Nomenclatore Tariffario delle prestazioni di assistenza specialistica ambulatoriale.

SUPPLEMENTARY MATERIAL 2: DETAILS ON DIRECT NON-HEALTHCARE COSTS

| **Transport** | | |
| --- | --- | --- |
| Item | Cost (€) | Description |
| Airplane | 100.00 € | Average flight cost, assuming a 1,000 km distance (as reported by interviewed subjects) ^1^ |
| Ambulance | 175.00 € | Cost inflated to 2019^2,3^ |
| Bus | 1.06 € | Average between highest and lowest ticket prices in Italy^4^ |
| Car/motorbike | 0.38 € | Average cost/km^5^ |
| Subway | 1.75 € | Average between metropolitan areas ticket prices^6,7^ |
| Taxicab | 4.26 € | Average between metropolitan areas tariffs; includes both fixed tariff and per km tariff^8,9^ |
| Train | 3.39 € | RFI tariffs inflated to 2019^3,10^ |
| **Other non-healthcare costs** | | |
| Item | Cost (€) | Description |
| Driving licence | 133.33 € | Average between driving licence renewal costs^11–13^ |
| **Paid assistance at home** | | |
| Item | Cost (€) | Description |
| Home aid, hour salary | 6.52 € | Average of tariffs included in the National Contract^13^ |
| Nurse, hour salary | 17.80 € | Calculated from average net salary, assuming 230 working days a year and 8 working hours a day^15,16^ |
| Professional caregiver, hour salary | 28.14 € | Salary for full-time professional caregiver for not self-sufficient patients, calculated from monthly salary^17^ |

1. Skyscanner.it, https://www.skyscanner.it/ (accessed 20 November 2021).

2. Chini F, Farchi S, Camilloni L, et al. Health care costs and functional outcomes of road traffic injuries in the Lazio region of Italy. *Int J Inj Contr Saf Promot* 2016; 23: 145–154.

3. Indice dei prezzi al consumo per famiglie operai e impiegati Gennaio 1947 - Ottobre 2021. *Istat Rivaluta*, http://rivaluta.istat.it:8080/Rivaluta/ (accessed 24 November 2021).

4. Altroconsumo.it, https://www.altroconsumo.it/vita-privata-famiglia/viaggi-tempo-libero/.

5. GU Serie Generale n.317 del 22-12-2020 - Suppl. Ordinario n. 42.

6. ATM Milan website, https://www.atm.it/it/ViaggiaConNoi (accessed 20 November 2021).

7. ATAC Rome website, https://www.atac.roma.it/biglietti-e-abbonamenti (accessed 20 November 2021).

8. City of Milan official website, https://www.comune.milano.it/aree-tematiche/mobilita/taxi (accessed 20 November 2021).

9. City of Rome website, https://www.comune.roma.it/web-resources/cms/documents/tariffario_taxi_A.pdf (accessed 20 November 2021).

10. Arrigo U, Di Foggia G. Produzione, costi e performance delle principali reti ferroviarie dell’Unione Europea.

11. Noicompriamoauto.it, https://www.noicompriamoauto.it/pratiche-auto/costo-rinnovo-patente (accessed 20 November 2021).

12. Quotidianomotori.com, https://www.quotidianomotori.com/ (accessed 20 November 2021).

13. Automobile.it, https://www.automobile.it/magazine/pratiche-auto/burocrazia/pagina-1 (accessed 20 November 2021).

14. Contratto Collettivo Nazionale di Lavoro sulla Disciplina del Rapporto di Lavoro Dometico, https://assindatcolf.it/wp-content/uploads/2021/04/CCNL-15X21-Assindatcolf-2020.pdf (2020, accessed 20 November 2021).

15. Contoannuale. Il costo del lavoro pubblico, https://www.contoannuale.mef.gov.it/ (accessed 20 November 2021).

16. Calcolostipendionetto.it, http://www.calcolostipendionetto.it/ (accessed 22 July 2021).

17. Assistere.net, https://www.assistere.net/quanto-costa-una-badante/ (accessed 20 November 2021).
